# *Salmonella* finds a way: Metabolic versatility of *Salmonella enterica* serovar Typhimurium in diverse host environments

**DOI:** 10.1371/journal.ppat.1008540

**Published:** 2020-06-11

**Authors:** Savannah J. Taylor, Sebastian E. Winter

**Affiliations:** Department of Microbiology, University of Texas Southwestern Medical Center, Dallas, Texas, United States of America; Tufts Univ School of Medicine, UNITED STATES

## Introduction

Infection with nontyphoidal *Salmonella* strains, such as *Salmonella enterica* serovar Typhimurium (*S*. Typhimurium) commonly causes foodborne bacterial gastroenteritis. Studies on the pathogenesis of *S*. Typhimurium infection often focus on bona fide virulence factors. *S*. Typhimurium produces two distinct type III secretion systems that allow for the delivery of a sophisticated repertoire of effector proteins into host cells. These type III secretion systems are required for invasion of intestinal epithelial cells and subsequent replication inside professional phagocytes in the lamina propria and in deeper tissues. In immunocompetent individuals, detection of *S*. Typhimurium by the innate immune system results in a subacute, neutrophilic inflammatory response that confines the infection to the intestinal tract. In immunocompromised individuals, such as those with neutropenia, systemic dissemination and replication can occur. While we understand key strategies of how *S*. Typhimurium causes disease, we are only beginning to recognize the complex interplay of host and bacterial metabolism. Recent findings have revealed how *S*. Typhimurium adapts its diverse energy metabolism to mirror host metabolism and limit nutrient competition in various host niches.

## Intracellular *Salmonella* adapts its metabolism to M1- and M2-polarized macrophages

**Fig 1:**
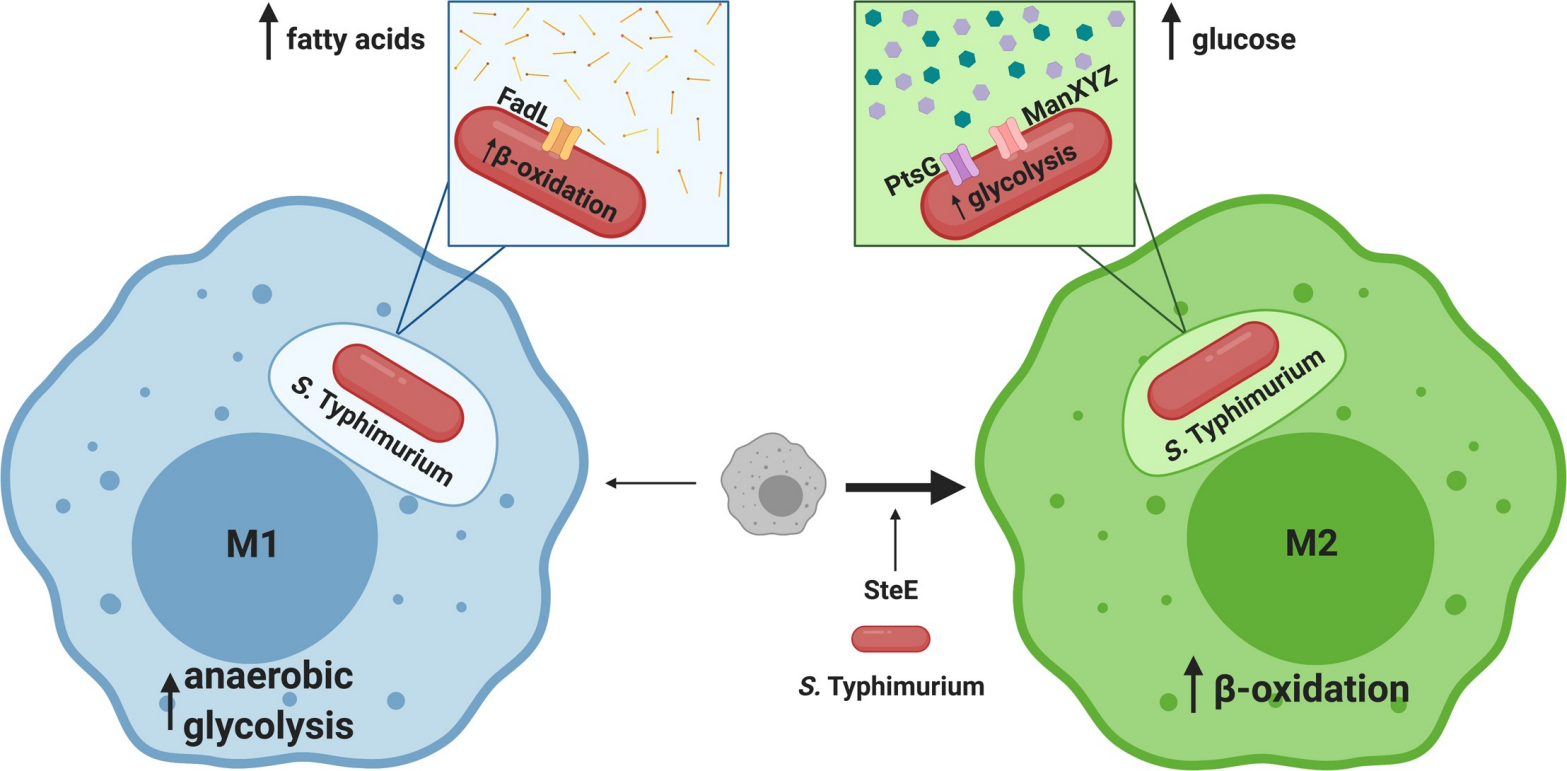
Intracellular *Salmonella* adapts its metabolism to M1- and M2-polarized macrophages. M1-type macrophages perform anaerobic glycolysis to avoid diverting oxygen from NADPH oxidase activity. *Salmonella* takes up fatty acids from the *Salmonella*-containing vacuole through the FadL transporter and degrades them through β-oxidation. The type III secretion system effector SteE drives M2 polarization. M2-type macrophages perform an oxidative metabolism. Inside M2-type macrophages, *Salmonella* utilizes sugars. Uptake of sugars is mediated by phosphotransferase systems (PtsG, ManXYZ) and possibly other transport systems.

In contrast to the self-limiting gastroenteritis observed in humans, *S*. Typhimurium establishes a systemic chronic infection in most mouse strains. *S*. Typhimurium survives and replicates in macrophages for weeks after infection, and the host immune system forms granulomas to contain pathogen spread. Most *S*. Typhimurium–infected macrophages belong to the M2 subtype [1,2] (**Fig 1**). M2 macrophages, commonly referred to as “anti-inflammatory” macrophages, promote wound healing and inhibit inflammation by producing IL-10 (interleukin 10) and TGF-β (transforming growth factor β) (reviewed in [3]). *S*. Typhimurium can also be found in other M2-like macrophage subtypes, such as granuloma macrophages, which share many characteristics with M2 macrophages but have distinct cell markers and functions [4]. Additionally, *S*. Typhimurium infects hemophagocytic macrophages, macrophages that have ingested nonapoptotic cells [5, 6]. Hemophagocytic macrophages express M2 markers and produce lower levels of inducible nitric oxide synthase (iNOS) [7].

*S*. Typhimurium induces polarization of infected macrophages [8] (**Fig 1**). Delivery of the bacterial effector SteE promotes a phenotypic switch to M2 polarization by driving noncanonical activation of STAT3 (Signal Transducer And Activator Of Transcription 3) signaling [8, 9]. SteE also induces a M2-like phenotype in infected granuloma macrophages [4]. This process is inhibited by TNFα (tumor necrosis factor α), which drives macrophage polarization toward an antimicrobial phenotype with higher iNOS activity [4]. In a process dependent on Toll-like receptor 4 (TLR4) signaling [6], *S*. Typhimurium also drives macrophages to increase hemophagocytosis or consumption of erythrocytes (red blood cells) and leukocytes (white blood cells) [5], creating a population of hemophagocytic macrophages with M2-like properties.

M2-type macrophages produce less antibacterial effector molecules, such as nitric oxide (NO) and reactive oxygen species, making them more permissive for *S*. Typhimurium replication. However, macrophage differentiation not only changes the cell’s role in the immune response, it also changes the cell’s metabolic program [3]. In persistently infected M2 macrophages, the transcription factor PPARδ (peroxisome proliferator-activated receptor δ) is activated [2]. PPARδ drives a lipid oxidation metabolism in the M2 macrophages, which results in more glucose available in the cell [2]. *S*. Typhimurium in infected M2 macrophages uses this glucose and other simple sugars to replicate [2, 10], presumably in a fermentative type of metabolism (**Fig 1**). In each of these M2-like macrophages, intracellular *S*. Typhimurium engineers a more permissive environment within its host macrophages with less exposure to antimicrobials. At the same time, *S*. Typhimurium responds to the metabolic reprogramming that accompanies polarization by relying on a glycolytic metabolism, while the macrophage uses mostly β-oxidation [2, 10, 11].

Despite their antibacterial function, M1 macrophages can also provide a site for *S*. Typhimurium replication [12] (**Fig 1**). M1 macrophages, also known as “pro-inflammatory” macrophages, produce large quantities of antimicrobials, such as reactive nitrogen species [3]. In contrast to M2 macrophages, M1 macrophages mainly carry out an anaerobic glycolytic metabolism [3]. Instead of competing with the host for nutrients, *S*. Typhimurium responds to the host’s glycolytic metabolic program by oxidizing lipids [12]. Lipid transport, β-oxidation, and glyoxylate shunt genes are required for *S*. Typhimurium to effectively replicate in M1 macrophages and spread to systemic sites [12]. Though *S*. Typhimurium counteracts many of the host’s defenses, exposure to NO in infected M1 macrophages still alters *S*. Typhimurium metabolism. The tricarboxylic acid cycle (TCA) cycle relies on iron–sulfur cluster-containing enzymes, which are very sensitive to damage by NO [13]. Diminished activity of these enzymes results in a transient methionine and lysine auxotrophy due to decreased production of succinyl-CoA, a metabolic intermediate in both the TCA cycle and amino acid biosynthesis [13]. Even within antimicrobial M1 macrophages, *S*. Typhimurium survives using its metabolic versatility to mirror the host’s metabolic program.

## *S*. Typhimurium utilizes inflammation-derived electron acceptors and exploits host energy metabolism

The gut microbiota protects the intestinal lumen from invasion by enteric pathogens, a phenomenon termed colonization resistance. To overcome colonization resistance, *S*. Typhimurium induces mucosal inflammation. Inflammation changes nutrient availability in the large intestine, thus allowing *S*. Typhimurium to outgrow the resident commensal microbiota and enhancing host transmission by the fecal–oral route [14, 15]. *Salmonella* populations in the intestinal lumen are heterogeneous [16]; a subpopulation invades the host mucosa and incites inflammation, while a second population replicates in the lumen away from the frontlines of the immune system.

**Fig 2:**
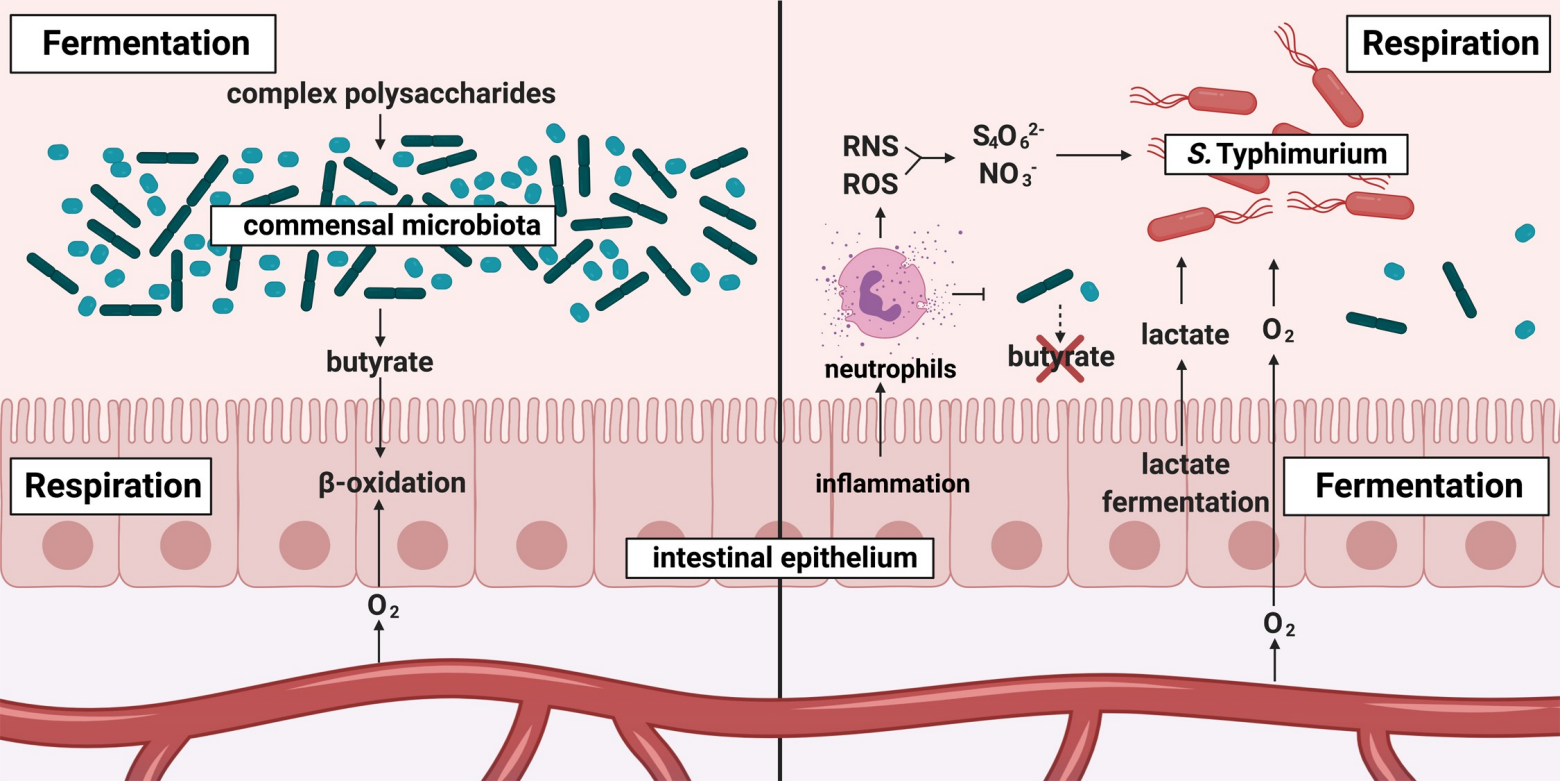
*S*. Typhimurium utilizes inflammation-derived electron acceptors and exploits host energy metabolism. During homeostasis (left panel), microbial fermentation of fiber results in the accumulation of short chain fatty acids, such as butyrate. Butyrate instructs intestinal epithelial cells to perform β-oxidation. This oxidative host metabolism depletes oxygen at the epithelial interface. During *Salmonella* infection (right panel), transmigrating neutrophils introduce RNS and ROS, which give rise to tetrathionate (S_4_O_6_^2-^) and nitrate (NO_3_^-^) in the lumen. Furthermore, inflammation depletes butyrate-producing bacteria, and the intestinal epithelium shifts to lactate fermentation. Lack of local oxygen consumption results in oxygen diffusing into the otherwise anaerobic gut lumen. Oxygen, tetrathionate, and nitrate are used by *Salmonella* as terminal electron acceptors to support an oxidative central metabolism. An oxidative metabolism allows for the efficient degradation of poorly fermentable carbon compounds, such as host-derived lactate. ROS, reactive oxygen species; RNS, reactive nitrogen.

The intestinal epithelium constantly interacts with gut bacteria, both in homeostasis and during enteric infection. These interactions include reciprocal metabolic pathways, where the epithelium consumes metabolites produced by the microbes and vice versa (**Fig 2**). During homeostasis, the microbiota consists mostly of obligate anaerobic bacteria from the orders Clostridiales and Bacteroidales. Members of Clostridiales, particularly *Clostridium* groups IV and XIVa, produce the short chain fatty acid butyrate [17] (**Fig 2**). Butyrate is the preferred carbon source for colon epithelium, used as a substrate for β-oxidation [18]. β-oxidation consumes most of the oxygen coming from the blood stream, maintaining physiological hypoxia in the gut lumen, which is ideal for the obligate anaerobic microbiota [19, 20]. *Salmonella*-induced inflammation depletes the butyrate-producing members of the microbiota [21] (**Fig 2**). In the absence of butyrate, the host epithelium switches its metabolism to anaerobic lactate fermentation [18]. Since lactate fermentation does not consume oxygen, oxygen from the blood stream diffuses into the gut lumen [19, 21]. *S*. Typhimurium, a facultative anaerobic bacterium, uses oxygen in the inflamed gut to outgrow the commensal microbiota through respiration [21]. In addition to more oxygen, the epithelial switch to lactate fermentation releases more lactate into gut lumen [22]. *S*. Typhimurium uses this lactate as a carbon source with its L-lactate dehydrogenase enzyme encoded by the gene *lldD* [22]. *S*. Typhimurium lactate utilization is coupled with oxygen respiration [22]. In the example of lactate, *S*. Typhimurium exploits lactate and oxygen, the products of an epithelial switch from respiration to fermentation, to feed its own respiratory metabolism and consequently outgrow the commensal anaerobic microbiota.

Early during infection, *Salmonella* requires fumarate reductase activity, suggesting that *S*. Typhimurium relies on a branched TCA cycle, in which the reductive branch assists with maintaining redox homeostasis [23]. At later time points, *S*. Typhimurium capitalizes on products of the innate immune response during infection. The inflammatory response induced by *Salmonella* involves transmigration of neutrophils into the gut lumen, where these immune cells release reactive oxygen species and reactive nitrogen species (**Fig 2**). These species indiscriminately react with host tissues, the resident microbiota, and metabolites available in the gut lumen. These metabolites include thiosulfate (S_2_O_3_^2-^), which is produced by the host epithelium to defuse the toxic microbiota byproduct hydrogen sulfide (H_2_S) [24, 25]. During an oxidative burst, thiosulfate reacts with reactive oxygen species to produce tetrathionate (S_4_O_6_^2-^) [26]. Furthermore, reactive nitrogen species decay into nitrate. Both tetrathionate and nitrate can be used by *S*. Typhimurium as an electron acceptor for anaerobic respiration [26, 27]. Respiration supports a full, oxidative TCA cycle [28] and the utilization of poorly fermentable carbon sources, such as succinate, lactate, 1,2 propanediol, and ethanolamine [22, 28–30]. In this example, *S*. Typhimurium uses its diverse respiratory metabolism to utilize inflammation-derived electron acceptors and to outcompete obligate anaerobic commensals.

## Conclusions

During infection, *S*. Typhimurium faces obstacles ranging from the gut microbiota to the immune system. Many aspects of *Salmonella* metabolism in the context of infection remain to be elucidated. However, the current literature reveals a pattern of reciprocal metabolism between *S*. Typhimurium and the host. *S*. Typhimurium uses its diverse metabolism to exploit local nutrient niches, outgrow resident microbiota, and persist within professional phagocytes.
